# Supplementary figures and images for: Patterns of overlapping habitat use of juvenile white shark and human recreational water users along southern California beaches
Source: PLoS One. 2023 Jun 2;18(6):e0286575. doi: 10.1371/journal.pone.0286575 (PMC10237640; doi:10.1371/journal.pone.0286575)

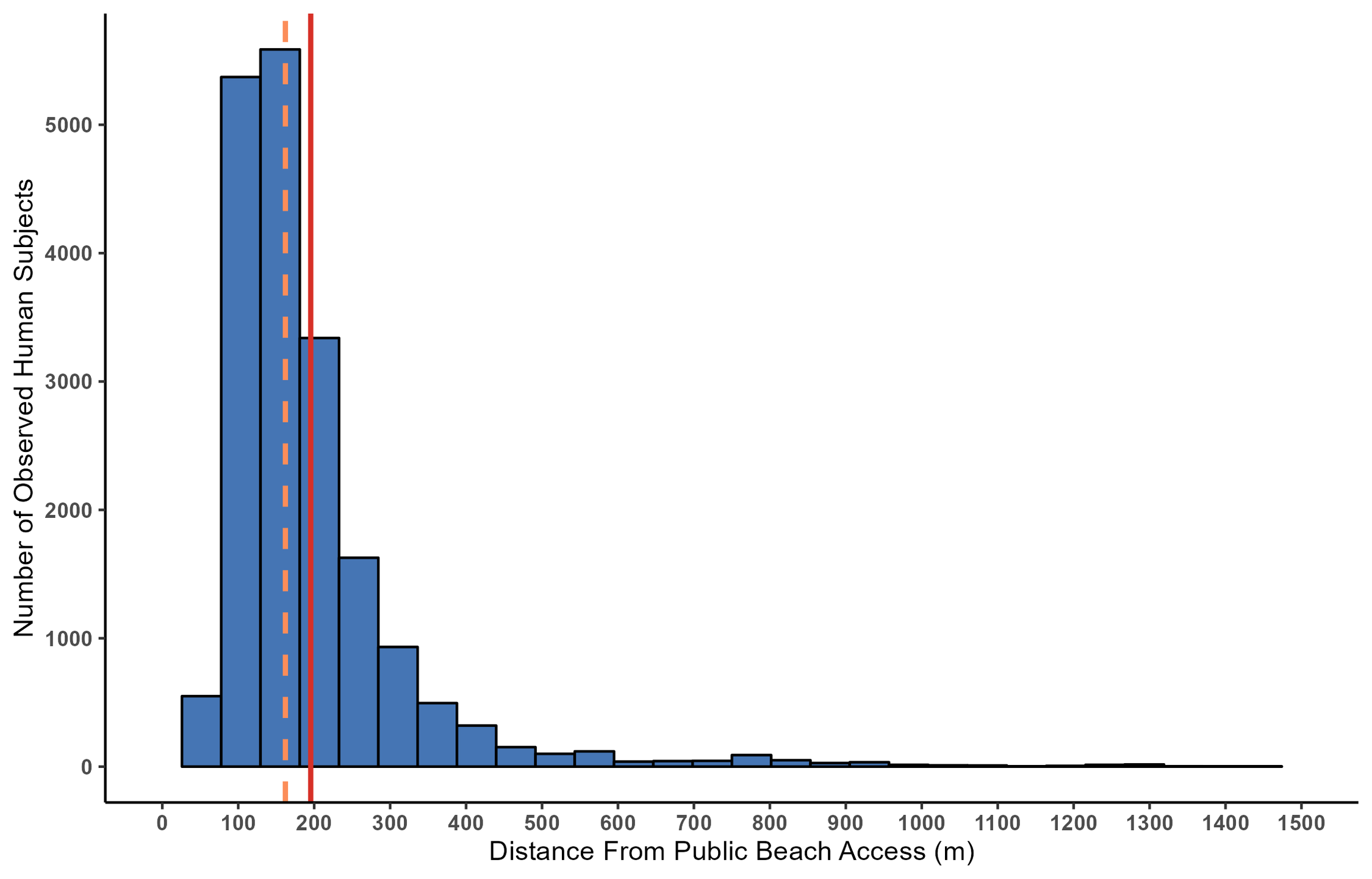

Supplement: S1 Fig — The solid line defines the mean of the data, while the dashed line defines the median of the data. (TIF) [file pone.0286575.s001.tif]

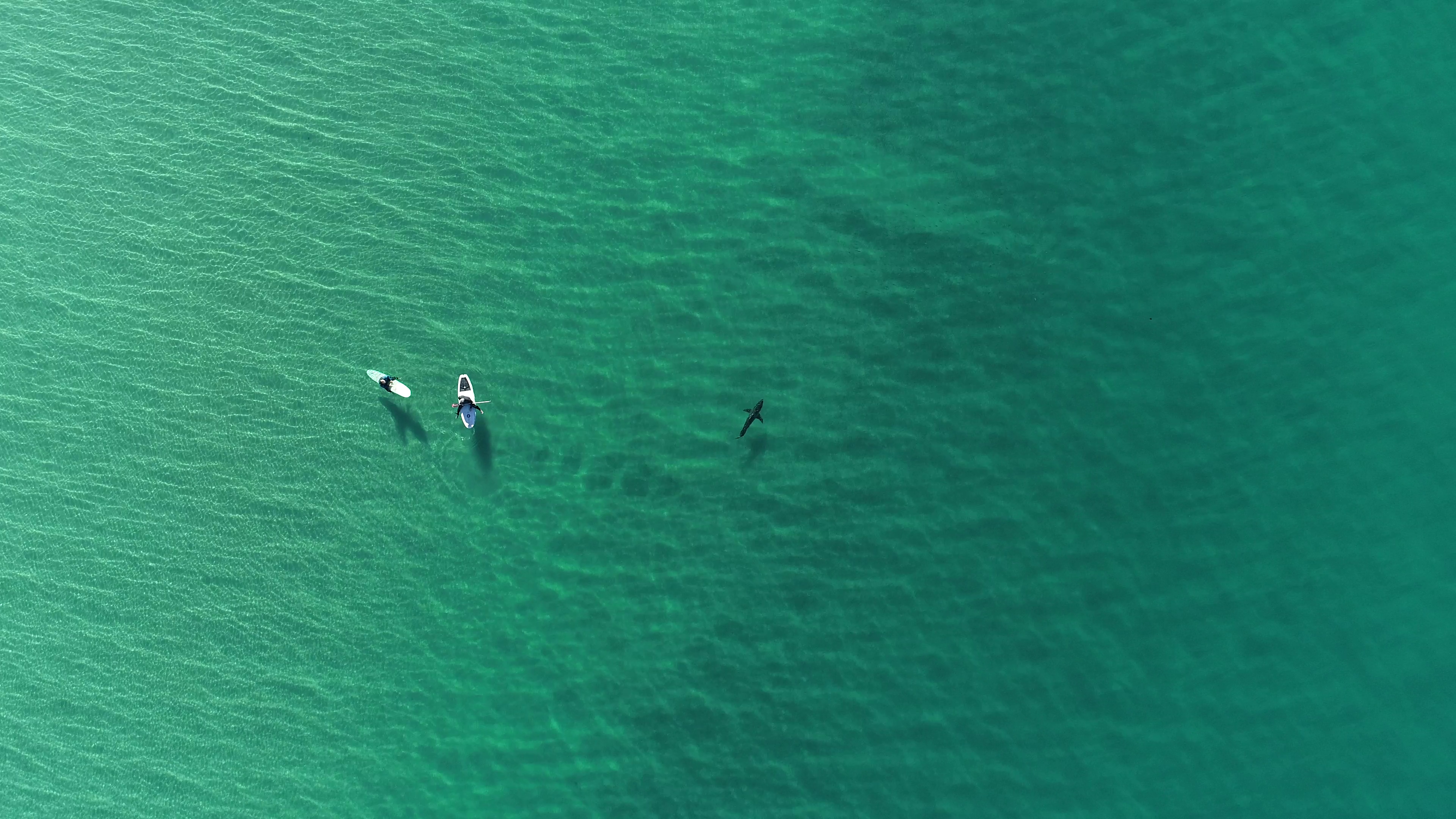

Supplement: S2 Fig — (PNG) [file pone.0286575.s002.png]
